# Association between Serum Levels of Interleukin-6 and Stage of Laryngeal Cancer

**Published:** 2015-05

**Authors:** Soheila Nikakhlagh, Nastran Ranjbari, Elaheh Khorami, Nader Saki

**Affiliations:** 1 *Hearing & Speech Research Center, Ahvaz Jundishapur University of Medical Sciences, Ahvaz, Iran. *

**Keywords:** Biomarker, Laryngeal Cancer, Squamous Cell Carcinoma

## Abstract

**Introduction:**

The purpose of this study was to investigate the relationship between serum levels of interleukin-6 (IL-6) and the severity and extent of squamous cell carcinoma (SCC) of the larynx based on stage of tumor progression and histological grade.

**Materials and Methods:**

All patients with a diagnosis of laryngeal cancer who underwent laryngoscopy and biopsy while hospitalized in Imam Khomeini Hospital in Ahvaz were enrolled. Tumor stage was calculated based on the TNM system, and divided into early (stage 1,2) or advanced stage (stage 3,4). In addition, patients were divided into low-grade (well differentiated) or high-grade (moderate and poorly differentiated) groups based on pathology reports from biopsy specimens. Several healthy volunteers were also enrolled as the control group. After collecting the blood samples, quantitative serum levels of IL-6 were measured (pmol/L) using IL-6 kits (Bender MedSystem, Germany). Results for quantitative variables are presented as mean and standard deviation and qualitative variables as percentages. Mann-Whitney, Kruskal-Wallis and Pearson’s chi square tests were used for statistical analyses. P<0.05 was considered statistically significant. Statistical analysis of data was performed using SPSS version 13.

**Results:**

Thirty-eight patients (82.6%) were male and eight patients (17.4%) were female. IL-6 serum level was 28.8±4.7 pmol/L in the patient group and 2.64±2.88 pmol/L in the control group (P=0.0001). The serum level of IL-6 was 7.27 ± 5.31 pmol/L in early-stage patients and 54.43 ± 6.06 pmol/L in advanced-stage patients (P<0.0001). IL-6 levels increased significantly with increasing N (according to TNM) (P=0.002). Levels of IL-6 in patients with metastasis were significantly higher than in the group without metastasis (P=0.024). Moreover, IL-6 levels increased significantly with increasing local tumor spread (T) (P<0.0001).

**Conclusion:**

This study shows that IL-6 is a gender-independent factor, serum levels of which are higher in patients with laryngeal SCC than in normal subjects. The results of this study also show that serum levels of this cytokine increase significantly with progression of this malignancy.

## Introduction

Laryngeal cancer is the second most prevalent cancer in the upper respiratory tract, affecting 11,000 individuals in the United States each year. Approximately 85–95% of laryngeal cancers are squamous cell carcinomas (SCC). Successful treatment requires accurate and timely diagnosis, staging and appropriate assessment of the patient. Laryngeal tumor staging is performed based on the sixth edition of the TNM system, published by the American Joint Committee on Cancer/Union for Internationals Cancer Control (AJCC/ UICC) (1).

Tumor grading is also expressed based on the pathologist’s report of biopsy specimens and according to cell differentiation (well, moderate, or poorly differentiated) (1).

Several factors affect the prognosis of patients with laryngeal cancer, including clinical stage and histological forms. Recent studies have examined genetic factors and biomarkers, in which the role of different interleukins has been described (2).

Interleukin-6 (IL-6) levels increase in many inflammatory diseases and malignancies. This cytokine which has multiple functions, and is involved in the differentiation of B lymphocytes into plasma cells. Studies have shown that IL-6 level is significantly associated with tumor invasion level, disease severity, and metastasis (3). IL-6 in conjunction with IL-1 and tumor necrosis factor-alpha (TNF-α) is one of the most important cytokines inducing acute phase inflammatory responses. This cytokine is secreted by B and T lymphocytes, macrophages, fibroblasts, keratinocytes, and other cells. In addition to the acute phase response, IL-6 also reinforces chronic phase inflammation, providing an ideal environment for the growth of tumors. On the other hand, IL-6 is also secreted by cancerous cells and plays a role in regulating proliferation and apoptosis (4). IL-6 activates Janus kinase (JAK) after binding to its receptor, where subsequent phosphorylation of the signal transducer and activator of transcription (STAT) occurs. The phosphorylated STAT gene is transferred into the nucleus and activates target genes such as vascular endothelial growth factor (VEGF) and rho which increase the rate of tumor invasion (5,6). 

The purpose of this study was to investigate the relationship between serum levels of IL-6 and the severity and extent of the disease based on tumor stage and histological grade in order to explore use of this biomarker as a prognostic factor and tumor marker. In the case of a relationship being identified, immunologic approaches could be used for the treatment of patients in the future.

## Materials and Methods

Forty-six patients with laryngeal cancer who were hospitalized and underwent biopsy in Imam Khomeini Hospital of Ahvaz between October 2012 and March 2013 were enrolled. Those previously treated with chemoradiation, patients who were using immunosuppressive drugs for any reason and patients with malignancies in other sites (except for laryngeal tumor metastases) were excluded. In all patients, video laryngoscopy, complete clinical examination, complete examination during laryngoscopy and biopsy, chest X-ray, computed tomography (CT) scan of the neck with contrast and abdominal sonography were performed and tumor stage was determined based on the TNM system. Patients were divided into two groups: early stage (stage 1,2) or advanced stage (stage 3,4). Moreover, based on the pathology reports of biopsy specimens, patients were divided into low-grade (well differentiated) or high-grade (moderate and poorly differentiated) groups. Other patient data such as age and gender were recorded on the questionnaire form along with the above information. Blood samples were then taken from all patients and collected in sterile tubes with lids and were immediately transferred to the laboratory for serum separation. In order to estimate the serum levels of IL-6 in normal subjects, samples were also taken from a number of non-patient volunteers. Then the sera were stored in a freezer at −20 °C. Melting and refreezing of the samples was prevented during storage. After collecting the samples, quantitative serum levels of IL-6 were measured (pmol/L) according to the enzyme-linked immunosorbent assay (ELISA) method using IL-6 kits (Bender MedSystem, Germany). After preparing the diluted solutions and adding substrate and stop solution, the levels of IL-6 were obtained using a spectrophotometer based on the absorption rate and a comparison with standard solutions. Results for quantitative variables are expressed as mean and standard deviation (mean ± SD) and as percentages for qualitative variables. Mann-Whitney, Kruskal-Wallis and Pearson’s chi square tests were used for statistical analysis. P<0.05 was considered to be statistically significant. Statistical analysis of data was performed using SPSS version 13. The Ethics Committee number is AJUMS. REC. 1393.68.

## Results

In this study, 63 individuals were examined; 46 of whom were patients with laryngeal cancer and 17 of whom were in the control group. Thirty-eight patients (82.6%) were male and eight (17.4%) were female. The age of patients was 63.78 ± 11.19 years. Serum IL-6 level was 28.8 ± 4.7 pmol/L in the patient group (range, 96–182.81 pmol/L) and 2.64 ± 2.88 pmol/L (range, 0.16–9.9 pmol/L) in the control group. Given the non-normal distribution of IL-6 levels, the Mann-Whitney statistical test was used to determine that mean IL-6 levels in patients with laryngeal carcinoma were significantly higher than those in the control group (P= 0.0001).

Of the 46 patients with laryngeal cancer, 25 (54.3%) were in the early-stage group and 21 (45.7%) were in the advanced-stage group. IL-6 levels were 7.27±5.31 pmol/L in the early-stage group and 54.43 ± 6.06 pmol/L in the advanced-stage group (P<0.0001) (Table. 1).

**Table 1:** IL-6 level according to stage of disease

**Lymph node involvement**	**Mean**	**SD**	**P-value**
Early	7.27	5.31	<0.0001
Advanced	49	56.5	
Metastatic	106	101.3	
			

IL-6 levels were 28.18±4.8 pmol/L in male patients and 31.72±4.51 pmol/L in female patients. No significant difference was observed between the two sexes in terms of IL-6 levels (P=0.653). 

Tumors were low grade in 24 (52.2%) patients and high grade in 22 (47.8%) patients. Statistically, the difference between the two tumor grades was at a borderline level (P = 0.057) (Table. 2).

**Table 2:** Levels of IL-6 according to tumor grade

**Tumor grade**	**Mean**	**SD**	**P-value**
Low grade	15.1	1.9	0.057
High grade	43.7	6.2	
			

The most common type of tumor in this study was glottic SCC, which was observed in 32 (69.6%) cases. No significant difference was observed between different tumor type and level of IL-6 (P = 0.496) (Table. 3). 

**Table 3:** Frequency distribution of various types of laryngeal tumors and IL-6

	**Frequency**	**Frequency Percentage**	**Mean**	**SD**	**P-value**
SCC glottis	32	69.6%	25.7	4.4	0.496
SCC transglottic	5	10.9%	50.2	7.4	
SCC subglottic	1	2.2%	7.18	-	
SCC supraglottic	7	15.2%	33	4.5	
Invasive SCC	1	2.2%	10.3	-	
Total	46	100%			
					

Distant metastases were observed in four (8.7%) patients. Mean ± SD IL-6 level was 72.1±7.2 pmol/L among this group, compared with 24.6±4.2 pmol/L in the group without distant metastases (P= 0.024). 

In terms of local tumor spread, most patients (70%) were T2 or T3. IL-6 levels increased significantly with increasing local tumor spread only (Table. 4). 

**Table 4:** IL-6 and tumor extension

**Tumor extension**	**Frequency**	**Frequency percentage**	**Mean**	**SD**	**P-value**
T1	8	17.4%	6.6	6.7	P<0.0001
T2	17	37%	7.5	4.7	
T3	15	32.6%	4.7	5	
T4	6	13%	88.7	7.4	
					

In terms of lymph node involvement, 26 (69.6%) cases were N0, 12 (26.1%) cases were N1, and two (4.3%) cases were N2. No patient was observed with N3. There was a significant difference between different levels of lymph node involvement and levels of IL-6 (P=0.002) (Table. 5).

**Table 5:** IL-6 level and lymph node involvement

**Lymph node involvement**	**Frequency**	**Frequency percentage**	**Mean**	**SD**	**P-value**
N0	32	69.6	16.3	3.2	0.002
N1	12	26.1	49.1	5.4	
N2	2	4.3	105.8	108.8	
					

## Discussion

Head and neck squamous cell carcinoma (HNSCC) is the eighth most common cancer worldwide and accounts for nearly six percent of all cancers (7). The larynx is the most common site of HNSCC. In Iran, as in other places of the world, the most common malignant tumor of the larynx is the SCC (8,9). The most common age of onset of this tumor is 60 years in Iran, and the male-to-female ratio is 9.5 to 1. In Iran, tumors located in the supraglottic region seem to be more common than those in the glottic area (10).

IL-6 is a cytokine with diverse functions. In conjunction with IL- 1 and TNF- α, it is one of the most important cytokines involved in inducing acute phase inflammatory responses. This cytokine also enhances the chronic phase of inflammation, providing an ideal environment for tumor growth (5,6).

Few studies have been conducted on the role of interleukins in whole head and neck carcinomas. Although the relationship between IL-6 and cancer has been extensively studied, the role of the production of this interleukin in the microenvironment of the tumor which progresses via malignant cells and tumoral infiltrated lymphocytes and macrophages is still controversial (11). Generally, it appears that early diagnosis or prevention of this cancer can be the most effective factor in improving patient outcomes. The absence of warning clinical signs for most HNSCCs increases the importance of identifying a biomarker with good sensitivity and specificity for screening patients at risk. So far, a number of molecular markers have been identified for the diagnosis of tumors, with varying degrees of sensitivity and specificity. IL-6 is a multifunctional cytokine which plays a pivotal role as a growth and differentiation factor in different cells such as hematopoietic progenitor cells, B-cells, T-cells, keratinocytes, neuronal cells, osteoclasts, and endothelial cells (12).

In different studies, IL-6 production has been reported in several tumors in humans such as esophageal SCC, multiple myeloma, renal cell carcinoma, glioblastoma, and lung carcinomas (13-17). Moreover, cycle of IL-6 - IL-6 receptor as been reported in a variety of tumors in several studies (14). In addition, the release of IL-6 has been reported by apoptotic T-cells in mice (18). These findings suggest that IL-6 produced by tumor cells enhances the survival and proliferation of cancer cells as a growth factor interacting with specific receptors on the surface membrane of cancer cells (13). Although recent studies have shown that an increased serum level of IL-6 is aligned with patient prognosis in various malignancies (19–22), the mechanism that causes this reaction in these diseases is still being discussed. The strongest hypothesis relating to the mechanism is that the increased level of IL-6 in cancer patients is due to excessive production from the tumor, and reflects the increase in serum levels of IL-6 as a biologic characteristic of the tumor However, the role of IL-6 in the growth of head and neck cancers is not yet well known. 

The present study aimed to investigate the relationship between serum levels of IL-6 and severity of laryngeal cancer. This study is among the few studies that examined the association of this cytokine with the severity of laryngeal malignancies. In this study, 46 patients with laryngeal cancer at different stages were studied. The mean age of patients was 63.8 years, and there were approximately five times as many men as women with this cancer. The mean age and gender prevalence in this study is consistent with other studies in this field (23).

The results of the present study primarily showed that the serum levels of IL-6 in patients with laryngeal cancer are significantly higher than in normal subjects in the control group. In a 2013 study conducted by Hao *et al.*, patients with laryngeal squamous cell cancer (LSCC) had signiﬁcantly higher levels of IL-6 compared with normal subjects, similar to the present study (24). However, in a 2009 study by Dae-Young Hong *et al.* investigating a number of biomarkers in patients with HNSCC, no significant difference was observed between the serum levels of IL-6 in patients with malignancy compared with the control group, indicating higher specificity of this cytokine in LSCC. 

The present study showed that IL-6 levels increased dramatically with progression of malignancy. The mean IL-6 level in early-stage patients was 7.27 pmol/L and 54.43 pmol/L in advanced-stage patients. This mean IL-6 level was significantly higher in advanced stages compared with early stages. In studies conducted in association with HNSCC, similar results to the present study were observed. For instance, a study by Riedel *et al.* in 2005 showed that IL-6 levels increased with increasing severity of disease (25). In a study conducted by Hao *et al.* regarding the diagnostic value of IL-6 and IL-8 in LSCC, it was determined that serum levels of IL-6 are directly correlated with metastasis to lymph nodes, local tumor spread, and clinical stage of tumor (P<0.05). Moreover, multivariate analysis showed that the serum level of IL-6 is an independent predictor for specific survival of LSCC (24). The Hao study was the closest to the present study, the results of which were also largely similar to our results. In another study by Duffy in 2007 in the United States in HNSCC patients, it was shown that the recurrence rate is higher in patients with higher levels of interleukin (P=0.002) and the survival rate is lower (3). Regarding the evaluation of serum levels of cytokines in patients with laryngeal cancer, a study was conducted in Southwest of Iran in levels of IL-10 in patients with laryngeal cancer and the associated metastases. No significant differences were found between patients with local invasion of laryngeal cancer and patients without local invasion. Furthermore, no statistically significant correlation was observed between IL-10 gene expression and different stages of tumor (26). The results of this study compared with the present study suggest that IL-6 is a more specific cytokine in laryngeal malignancies, levels of which increase considerably both compared with normal subjects and in advanced stages of tumor. The results of the present study showed that IL-6 is a gender-independent factor whose serum level in patients with laryngeal SCC is higher than in normal subjects. Moreover, this study indicated that the serum level of this cytokine dramatically increases with the progression of malignancy, so that serum levels of IL-6 were significantly higher in advanced-stage patients compared with early-stage patients. Furthermore, patients with high-grade tumors, more lymph node involvement, distant metastases and higher levels of T classification had higher serum levels of IL-6.

## Conclusion

Laryngeal cancer is the second most common cancer in the upper respiratory tract. Finding new cellular and molecular methods for early detection of aggressive cases and timely treatment can reduce the associated mortality. According to the results of the present study, serum levels of IL-6 in patients were significantly associated with the severity of laryngeal cancer. Therefore, this biomarker can be used in the diagnosis and treatment of invasive cases. Further studies are needed to accomplish the goals described.
